# Exploration of Toxins from a Marine Annelid: An Analysis of Phyllotoxins and Accompanying Bioactives

**DOI:** 10.3390/ani14040635

**Published:** 2024-02-16

**Authors:** Ana P. Rodrigo, Inês Moutinho Cabral, António Alexandre, Pedro M. Costa

**Affiliations:** 1 Associate Laboratory i4HB Institute for Health and Bioeconomy, NOVA School of Science and Technology, NOVA University of Lisbon, 2829-516 Caparica, Portugal; imf.cabral@campus.fct.unl.pt (I.M.C.); at.alexandre@campus.fct.unl.pt (A.A.); 2 UCIBIO Applied Molecular Biosciences Unit, Department of Life Sciences, NOVA School of Science and Technology, NOVA University of Lisbon, 2829-516 Caparica, Portugal

**Keywords:** marine invertebrates, toxins, biotechnology, in silico analysis, homology matching, bioprospecting

## Abstract

**Simple Summary:**

Evolution provided animals with efficient predatory and defense mechanisms that may include the secretion of bioactive molecules, referred to as toxins, that disrupt physiological processes in their recipients. Owing to their reactivity and, often, relative specificity, toxins hold biotechnological interest, especially proteinaceous toxins, which may be more easily manipulated and synthetized in vitro. Here, we investigated putative cysteine-rich neurotoxins from the marine worm *Eulalia* (‘phyllotoxins’) and other bioactive proteins by isolating their full coding sequence and analyzing their potential mode of action using computational methods. The findings suggest that some of these toxins can be highly bioactive and that their specificity may render them interesting for further investigation as painkillers, anticoagulative drugs, or even as a method of enhancing the effect of other drugs.

**Abstract:**

Proteinaceous toxins are peptides or proteins that hold great biotechnological value, evidenced by their ecological role, whether as defense or predation mechanisms. Bioprospecting using bioinformatics and omics may render screening for novel bioactives more expeditious, especially considering the immense diversity of toxin-secreting marine organisms. *Eulalia* sp. (Annelida: Phyllodocidae), a toxin bearing marine annelid, was recently shown to secrete cysteine-rich protein (Crisp) toxins (hitherto referred to as ‘phyllotoxins’) that can immobilize its prey. By analyzing and validating transcriptomic data, we narrowed the list of isolated full coding sequences of transcripts of the most abundant toxins or accompanying bioactives secreted by the species (the phyllotoxin Crisp, hyaluronidase, serine protease, and peptidases M12A, M13, and M12B). Through homology matching with human proteins, the biotechnological potential of the marine annelid’s toxins and related proteins was tentatively associated with coagulative and anti-inflammatory responses for the peptidases PepM12A, SePr, PepM12B, and PepM13, and with the neurotoxic activity of Crisp, and finally, hyaluronidase was inferred to bear properties of an permeabilizing agent. The in silico analysis succeeded by validation by PCR and Sanger sequencing enabled us to retrieve cDNAs can may be used for the heterologous expression of these toxins.

## 1. Introduction

Whether they are delivered as components of complex venoms directed to specific molecular targets or as defensive substances to keep potential predators and foulants at bay, the production of toxins is a fundamental ecological trait that is continuously shaped by adaptive pressure. Apart from different interpretation and definitions of what constitutes a toxin, a poison, or a venom, investigating the biochemical properties of such bioactives or their mixtures is paramount to understand not only how a species adapted to their surroundings via chemical warfare but also provide humans with tools to render them into potential biotechnological assets [1,2]. Indeed, integrating ecology, genetic engineering, and areas of life sciences such as microbiology, physiology, and biomedicine can lead to the exploitation and even modification of biochemical assets such as toxins to produce bioactives that are optimized for a desired goal such as a painkiller, novel antibiotic, or an eco-friendly pesticide [3]. Even though toxins are, per se, highly prized bioactives for this purpose due to their inherent bioactivity with varying specificity, screening biodiversity for toxins and toxin-secreting organisms is a painstaking challenge that calls for novel approaches to systematize the research and discovery process. With this respect, venomics, i.e., addressing the ecology and evolution of venoms using omics techniques, gave a great boost to the discovery of molecules with biotechnological potential [4]. Moreover, the possibility of synthesizing these molecules in vitro (e.g., recombinant proteins) and eventually scaling up their production is also key for the preservation of natural resources [5]. The synergism between continuously advancing computational tools, databases for compounds, and processes now seems to be an unavoidable component in marine bioprospecting.

Assisted by the oceans’ immense biodiversity, bioprospecting for marine bioactives can reduce the riskiness and expensiveness of the processes required for developing synthetic compounds [6,7,8]. Even though the main focus has been set onto secondary metabolites, the proteinaceous nature of animal bioactives like many toxins can become an important biotechnological asset due to the safe handling, the easiness of peptidic structure modification, high selectivity, and the low costs normally involved, especially if dealing with heterologous expression through DNA recombinant methods using convenient microbiological models, which is a massive advantage for scaling up at the industrial scale (reviewed by Pennington et al. [9]). Indeed bioactive marine peptides exhibit various biological activities, such as antiviral, antimicrobial, antifungal, antiproliferative, antioxidant, anticoagulant, antihypertensive, anticancer, antidiabetic, antiobesity, and calcium-binding properties (reviewed by Giordano et al. [5]). A well-known example is *Conus*, a genus of mostly toxin-secreting gastropods that rapidly immobilize prey with conotoxins, which are cysteine-rich proteinaceous neurotoxins able to block neuronal ion channels. A recombinant conotoxin (ziconotide) has been turned into a drug (Prialt) for the treatment of severe chronic pain [10].

Marine annelids are taking the first steps in showing their biotechnological potential, albeit still not yet transformed into consumer end products. This is the case with ovothiol, a possible pheromone produced by males to attract females in the species *Platynereis dumerilii* and has the potential to treat infections or even serve as an anti-atherogenic (reviewed by Castellano and Seebeck [11]). In turn, hemoglobin derived from the marine annelid *Arenicola marina* presents as seemingly more efficient and safer for human use than mammalian forms [12]. Another example is *Glycera*, which has specialized venom glands [13] and whose toxins’ biotechnological potential was inferred using transcriptomics and bioinformatics to find potential molecular targets in the human druggable proteome [14]. *Eulalia* sp. (Annelida: Phyllodocidae), the subject of our current case study, is a species reported to have a wide distribution, from the north Atlantic to the Mediterranean Sea and all across the American Atlantic coast until central America, to which there are added reports from Alaska; however, this wide distribution is indicative of the possible overlapping of species or subspecies. In fact, the species reported as *Eulalia viridis* from the Atlantic coast of France, Portugal, and Spain were found to be most likely *Eulalia clavigera* [15,16], even though a complex should not be excluded at this stage. *Eulalia* sp. feeds mostly on barnacles, mussels, and other worms, even from the same species [17,18]. This worm, like other phyllodocids, makes use of its proboscis for collecting food through suction by using its strong axial musculature [19,20]. In addition, the proboscis secretes a mixture of toxins and other bioactives that use mucous as vehicle, within which a protein or group of proteins is present, referred to as ‘phyllotoxins’, which are potential cysteine-rich neurotoxins [21,22]. In the face of these preceding discoveries, the main aim of the present work is to identify and isolate the full coding sequence of phyllotoxins and other relevant bioactive proteins secreted by *Eulalia* sp. to enable their future heterologous expression for research and biotechnological purposes.

## 2. Materials and Methods

### 2.1. Transcriptome Analysis

To identify mRNAs coding for putative proteins in the proboscis, which is involved in toxin secretion and delivery, the body wall was taken as reference organ, as already described by Rodrigo et al. [21]. The transcriptomic data analyzed in the current work have been produced by a preceding work [21] and is freely accessible via the Gene Expression Omnibus (GEO), accession number GSE143954. In brief, after an RNA quality assessment (intact/partially degraded samples with RIN ≥ 7, input of ≥1 µg total RNA), samples were sequenced, and the transcriptome was assembled (*n* = 3 replicates for both proboscis and body wall, with 1 replicate per organ sequenced with high depth, 100 M reads, and the remaining with normal coverage, 20 M reads). Proboscis-specific transcripts overexpressed with coding regions were obtained and annotated by homology against protein domains from PFam v32.0 [23] using HMMER v3.1b2 [24].

### 2.2. Selection of Toxin Transcripts

The proteins that were previously shortlisted and identified as promising biotechnological targets [21] were selected for analysis, namely phyllotoxins, known as cysteine-rich secretory protein (Crisp); hyaluronidase; serine protease; peptidase M12A; peptidase M13; and peptidase M12B. Then, we selected the respective transcripts overexpressed in *Eulalia* proboscises with logFCs > 10 (i.e., with a significant differential gene expression between the proboscis and body wall). An initial sequence analysis was carried out on the multiple transcript variants of the toxins to exclude the transcripts without a representative coding region. The next step was to scan for homology matching against Swiss-Prot (version 2022-08-08) and NCBI’s non-redundant protein sequences database with BLASTP [25] (BLAST + version 2.13.0). The transcript selection criteria taken into account were the following: (i) an initial correspondence of the protein of interest’s characteristics with the sequence description showcased in the database; (ii) a high query cover (>70%) within the homologs and the transcript data having been analyzed; (iii) a low *e*-value (lower than 1 × 10^−30^), indicating a high level of similarity between the homolog matches and the transcript variants being screened; (iv) the quantitative overexpression of a specific variant in the proboscis when compared to the reference levels in the body wall. A transcript was selected per protein (unrelated to the ones selected in the previous work [21]).

### 2.3. RNA Collection

#### 2.3.1. Animal Collection

Adult *Eulalia* sp. with an undistinguishable maturation stage (≈120 mm total length and weighting ≈ 250 mg each) were collected from the West Coast of Portugal (38°41′42″ N, 09°21′36″ W) on an intertidal rocky beach. The animals were reared in the laboratory in a mesocosm environment, recreating their natural habitat (rocky mussel beds) while controlling the salinity, temperature, and photoperiod (35 ± 1, 16 ± 1 °C, and 10:16 h, respectively). Animals were fed with live mussels, one of the species’ favorite preys. Six specimens were used for the subsequent analyses.

#### 2.3.2. RNA Extraction

The worms were dissected for the excision of their proboscises and body walls. The extraction of the total RNA was performed according to the methods described in Rodrigo et al. [21]. In brief, portions of tissue were infiltrated with RNALater and an RNeasy Protect Mini Kit was used and coupled with in-column DNA digestion using an RNAase-free DNAase set (all from Qiagen, Hilden, Germany) following the manufacturer’s instructions. The quantification of the total RNA and initial quality assessment was performed using a Nanodrop 1000 spectrophotometer (Thermo Fisher Scientific, Waltham, MA, USA). Samples were stored at −80 °C until further analysis.

### 2.4. Sequence Isolation and Expression Analysis

The transcripts from the proboscises and body walls, from immature *Eulalia* sp., were selected from RNA-seq data previously obtained and validated by a polymerase chain reaction, as described in Rodrigo et al. [21]. In brief, cDNA was synthetized from the total RNA samples using a First-Strand cDNA Synthesis Kit (NZYTech, Lisbon, Portugal). Primers were designed using Primer Blast [26] and verified in silico using an Oligo Analyzer to amplify an expressed sequence tag (EST) for the selected genes (Table S1). After resolving the PCR products in an agarose gel, they were Sanger-sequenced in an ABI 3730 X1 sequencer 154 using a BigDye Terminator sequencing kit (both from Thermo Fischer Scientific, Waltham, MA, USA). The translated products were analyzed, and the sequence obtained for each gene confirmed and contrasted with the ones from the RNA-seq data.

The expression of the validated sequences of the proboscises was compared with the that of the body walls and confirmed by a reverse transcription polymerase chain reaction (RT-qPCR). New primers were selected (Table S2) to amplify the target sequences plus 18S as an internal control [14]. It was performed in a Corbett Rotor-Gene 6000 thermal cycler (QIAGEN, Hilden, Germany) using the NZY qPCR Green Master Mix (NZYTech). The program included an initial denaturation stage (95 °C, 10 min), followed by 45 cycles of denaturation (94 °C, 45 s), annealing (54 °C, 35 s), and extension (72 °C, 30 s). An expression analysis was performed using the 2^−ΔΔCt^ method [27] (Figure S1). A primer-melting analysis was also conducted to verify the specificity of the hybridization.

#### Individual-Gene Phylogenetic Analysis

Sequences from each protein were chosen to be scanned for homology against NCBI’s RefSeq and UNIPROT databases using Blast [28] for the best hits of each protein and according to the following clades: Mammalia, Arachnida, Cephalopoda, Annelida, Hymenoptera, Bivalvia, Reptilia, and Scorpionida. After alignment, the best model for each protein was selected according to the lowest Bayesian information criterion (BIC), as follows: the Whelan and Goldman (WAG) model with discrete gamma distribution (G) and evolutionarily invariable (I) for the peptidase M12A, the WAG+G model for serine protease, the Le Gascuel model (LG) plus G+I for the peptidase M13, and WAG+G+F (frequencies) for hyaluronidase and the peptidase M12B. Phylogenetic trees were produced (1000 bootstrap pseudoreplicates) for the sequences of each individual protein using maximum likelihood, following Tamura and Nei [29]. A sequence alignment and trees were produced using Mega X (version 10.1.8) [30]. The exception was made for Crisp, as the same sequence was used in Rodrigo et al. [21] for the phylogenetic analysis.

### 2.5. Sequences Analysis for Biotechnology Potential

*Homo sapiens* homologues (customized Swiss-Prot database containing only human proteins, version 2022-08-08, accessed at https://ftp.ncbi.nlm.nih.gov/blast/db/swissprot.tar.gz, on 10 August 2022) were found for the short-listed proteins using only annotated sequences from BLASTP (BLAST + version 2.13.0) [25] and were chosen based on their *e*-value, cover percentage, and subcellular location. Interactome and gene network analyses were conducted using the Human Reference Protein Interactome Mapping Project [31] (HuRI, accessed on 24 March 2022) and the Search Tool for the Retrieval of Interacting Genes/Proteins [32] (STRING, v12.0), respectively. In STRING, the confidence cut-off for interaction links between proteins was set at 0.400, and the functional enrichment (the approach used to extract biological knowledge from omics experimental results) was analyzed [33].

## 3. Results

**Figure 1:**
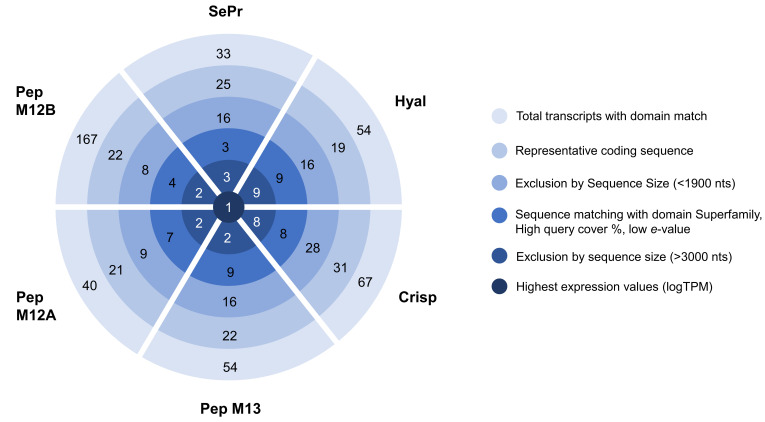
Identification of the transcripts of interest using raw RNA-seq data produced by Rodrigo et al. [21]. Subsequent narrowing process of the best transcript per proteinaceous toxin with biotechnological interest using several constraints.

The main toxins secreted by *Eulalia* sp., after being identified by Rodrigo et al. [21], were analyzed and narrowed down to one target transcript per toxin, as follows: cysteine-rich secretory protein or phyllotoxin (Crisp), hyaluronidase (Hyal), serine protease (SePr), peptidase M12A (PepM12A), peptidase M13 (PepM13), and peptidase M12B (PepM12B). The target transcripts were identified according to complete predicted coding sequence, sequence size, expression value, and homology matching, with their use for biotechnological purposes as the main goal (Figure 1).

### 3.1. Identification of Putative Toxins

For each selected transcript, the sequences were validated (Table S3), and their expression was confirmed by RT-qPCR (Figure S1). A phylogenetic analysis of the individual components was performed to assess which organism protein/enzyme had best homology-matching similarities with the ones present in *Eulalia*, thus enabling comparative toxinology and the gauging of the functionality of each individual molecule (Figure S2). The phylogenetic analysis verified the certainty of the proteins chosen due to the higher resemblances with transcripts identified from *Glycera* (retrieved from the work of Moutinho Cabral et al. [14]). Other species in the same branch were mainly from the phylum Mollusca, including known venomous organisms such as the ones from *Conus* genus, known to produce, for example, Hyal and PepM13. When the sequences were matched with the curated databases (Table 1), i.e., the full sequences from *Eulalia* sp. matched with full curated sequences, all the toxins were “secreted”, and some had their best match with proteins from venomous organisms (namely Crisp, Hyal, andPepM12B).

### 3.2. Human Homology Matching

Keeping in mind the potential biotechnological value of these putative proteins, in the absence of specific genomic resources for non-model organisms, we looked for human homologs of these toxins as a means to find target receptors within the human interactome (Table 2).

Human homologs were found for all the putative proteins, and similar *e*-values were shown between their best match and the human homolog in general. The homology matching gave the same family proteins in both cases (Table 1 and Table 2); however, even though all the toxins from *Eulalia* sp. have higher resemblance with secreted proteins (recall Figure S2), not all the homologs had a high match (mainly *e*-value) with secreted proteins from humans (like Hyal). Moreover, due to the similarities between genes and *e*-values, more than one best match was analyzed in most cases.

To identify the full potential of the selected toxins through homology matching against human proteins, the human interactome and network of protein–protein interactions were analyzed, when available, considering the best matches. Based on the protein–protein interactions, both databases revealed key proteins and/or pathways that highlight the potential of the selected proteins for biomedical applications. Moreover, there were some human homologs that could be identified in the HuRI platform and could provide more interaction information, like the human homologs of PepM12B and Crisp that interact with the VWF and GPR152, respectively. From STRING software (v12.0), more protein interactions were evidenced.

**Figure 2:**
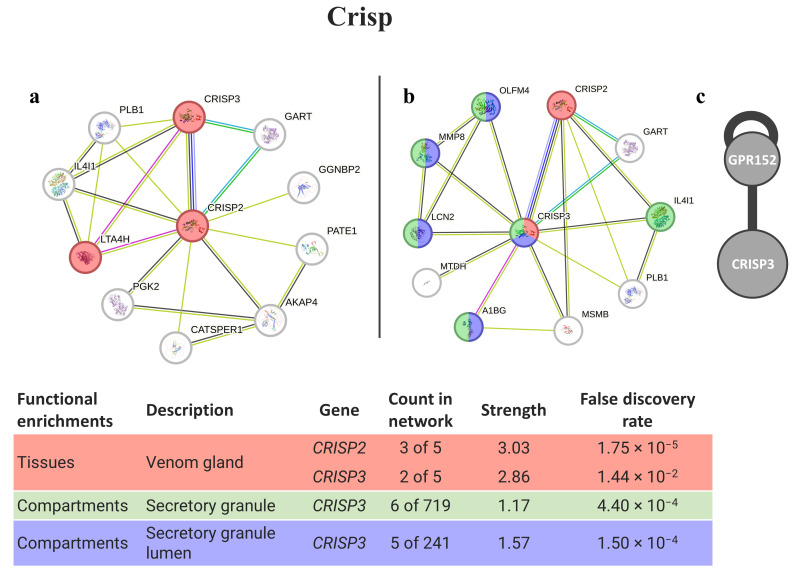
Crisp interaction pathways for the best homologs matches in humans. (**a**) Protein–protein interaction network obtained by STRING software for CRISP2; (**b**) protein–protein interaction network obtained by STRING software for CRISP3; (**c**) human interactome from the HuRI for CRISP3. The confidence cut-off for showing interaction links was set to medium (0.400). A1BG—alpha-1-B glycoprotein; AKAP4—A-kinase anchor protein 4; CATSPER1—cation channel sperm-associated protein 1; CRISP2—cysteine-rich secretory protein 2; CRISP3—cysteine-rich secretory protein 3; GART—trifunctional purine biosynthetic protein adenosine-3; GGNBP2—gametogenetin-binding protein 2; GPR152—G protein-coupled receptor 152; IL4I1—L-amino-acid oxidase; LCN2—neutrophil gelatinase-associated lipocalin; LTA4H—leukotriene A-4 hydrolase; MMP8—neutrophil collagenase; MTDH—protein LYRIC; OLFM4—olfactomedin-4; PATE1—prostate and testis expressed 1; PGK2—phosphoglycerate kinase 2; PLB1—phospholipase B1, membrane-associated.

Matching the phyllotoxin (Crisp) with potential human homologs identified CRISP3 and CRISP2 as the best matches (Figure 2). Both proteins may interact with themselves, and some variants can be found in the venom glands of animals. Moreover, several proteins from the CRISP3 network are present in secretory granules.

**Figure 3:**
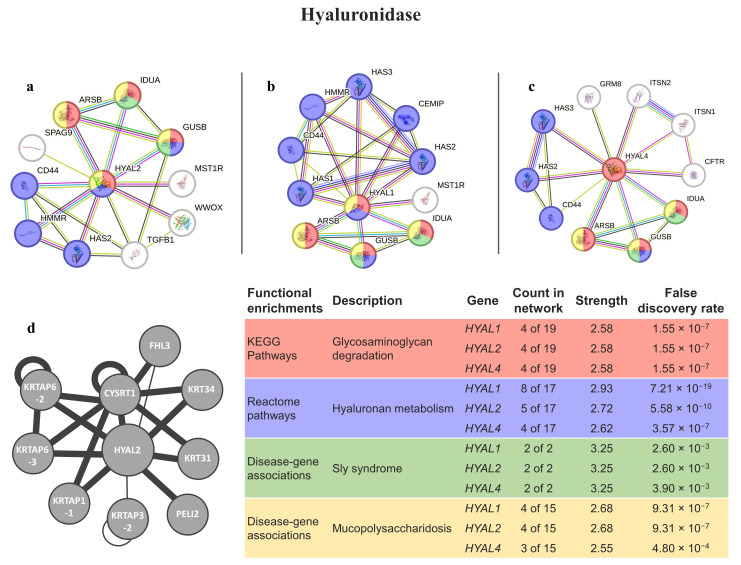
Hyal interaction pathways for the best homologs matches in humans. (**a**) Protein–protein interaction network obtained by STRING software for HYAL2; (**b**) protein–protein interaction network obtained by STRING software for HYAL1; (**c**) protein–protein interaction network obtained by STRING software for HYAL4; (**d**) human interactome from the HuRI for HYAL2. The confidence cut-off for showing interaction links was set to medium (0.400). ARSB—arylsulfatase B; CEMIP—cell-migration-inducing and hyaluronan-binding protein; CD44—CD44 antigen; CFTR—cystic fibrosis transmembrane conductance regulator; CYSRT1—cysteine-rich tail 1; FHL3—four-and-a-half LIM Domains 3; GRM8—metabotropic glutamate receptor 8; GUSB—beta-glucuronidase; HAS1—hyaluronan synthase 1; HAS2—hyaluronan synthase 2; HAS3—hyaluronan synthase 3; HMMR—hyaluronan-mediated motility receptor; HYAL1—hyaluronidase-1; HYAL2—hyaluronidase-2; HYAL4—hyaluronidase-4; IDUA—alpha-L-iduronidase; ITSN1—intersectin-1; ITSN2—intersectin-2; KRT31—keratin 31; KRT34—keratin 34; KRTAP3-2—keratin-associated protein 3-2; KRTAP1-1—keratin-associated protein 1-1; KRTAP6-2—keratin-associated protein 6-2; KRTAP6-3—keratin-associated protein 6-3; MST1R—macrophage-stimulating protein receptor alpha chain; PELI2—E3 ubiquitin-protein ligase pellino homolog 2; SPAG9—C-Jun-amino-terminal kinase-interacting protein 4; TGFB1—transforming growth factor beta-1 proprotein; WWOX—WW domain-containing oxidoreductase.

The best matches with hyaluronidases (HYAL1, HYAL2, and HYAL4) were found to be involved in glycosaminoglycan degradation. They were also found to be related to disease genes for sly syndrome and mucopolysaccharidosis (Figure 3). The only protein present in the HuRI is HYAL2, which is mostly related to keratin-associated proteins.

**Figure 4:**
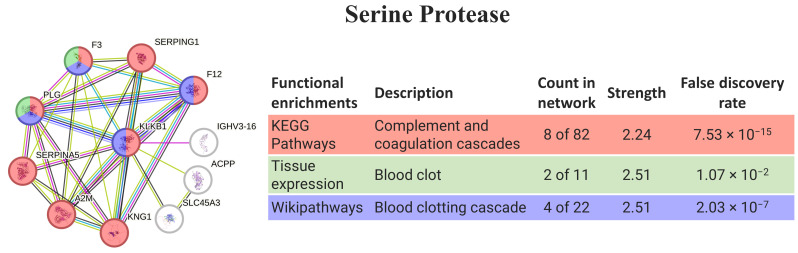
SePr interaction pathway for the best homolog match in humans using the protein–protein interaction network obtained by STRING software for KLKB1. Protein–protein interaction networks obtained by STRING software (colored networks) and the HuRI (grey network) for the best matching homologs in humans in the case of serine protease (KLKB1). The confidence cut-off for showing interaction links was set to medium (0.400). A2M—alpha-2-macroglobulin; ACPP—prostatic acid phosphatase; F12—coagulation factor XII; F3—tissue factor; IGHV3-16—probable non-functional immunoglobulin heavy variable 3-16; KLKB1—plasma kallikrein heavy chain; KNG1—kininogen-1; PLG—plasminogen; SERPINA5—plasma serine protease inhibitor; SERPING1—plasma protease C1 inhibitor; SLC45A3—solute carrier family 45 member 3.

The human homolog of serine protease (KLKB1) is directly involved in the immune complement, coagulation, and blood-clotting cascades (8 of 11 proteins). In fact, the biological processes more relevant to the pathway are related to coagulation, and a few are related to lectin pathways, thus they are connected with the defense mechanism (Figure 4).

**Figure 5:**
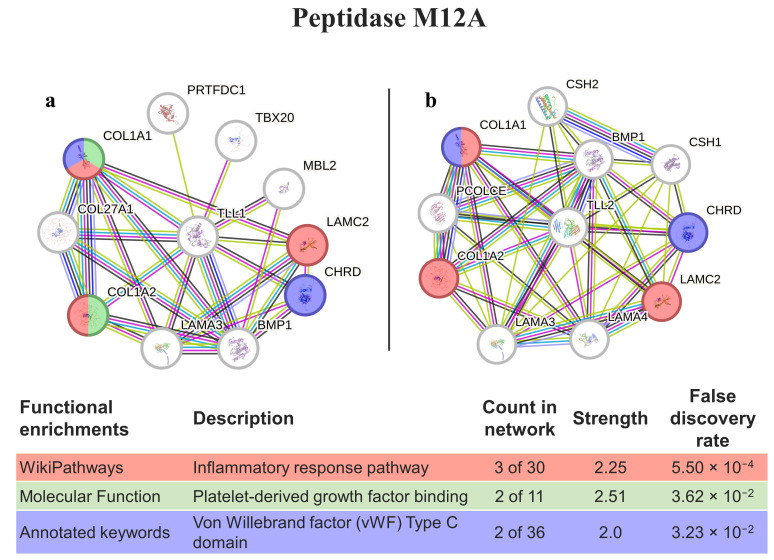
PepM12A interaction pathways for the best homolog matches in humans. (**a**) Protein–protein interaction network obtained by STRING software for TLL1; (**b**) protein–protein interaction network obtained by STRING software for TLL2. The confidence cut-off for showing interaction links was set to medium (0.400). BMP1—bone morphogenetic protein 1; CHRD—chordin; COL1A1—collagen alpha-1(I) chain; COL1A2—collagen alpha-2(I) chain; COL27A1—collagen alpha-1(XXVII) chain; CSH1—chorionic somatomammotropin hormone 1; CSH2—chorionic somatomammotropin hormone 2; LAMA3—laminin subunit alpha-3; LAMA4—laminin subunit alpha-4; LAMC2—laminin subunit gamma-2; MBL2—mannose-binding protein C; PCOLCE—procollagen C-endopeptidase enhancer 1; PRTFDC1—phosphoribosyltransferase domain-containing protein 1; TBX20—T-box transcription factor TBX20; TLL1—tolloid-like protein 1; TLL2—tolloid-like protein 2.

Peptidase M12A homology matching was performed with the genes responsible for expressing tolloid-like proteins (TLL1 and TLL2, Figure 5). When considering both interactomes, 6 out of the 10 proteins were the same and, in both cases, had proteins involved in the inflammatory response and von Willebrand factor (in common with ADAMTS13); however, the protein TLL1 had a molecular function platelet-derived growth factor binding that was not present in TLL2.

**Figure 6:**
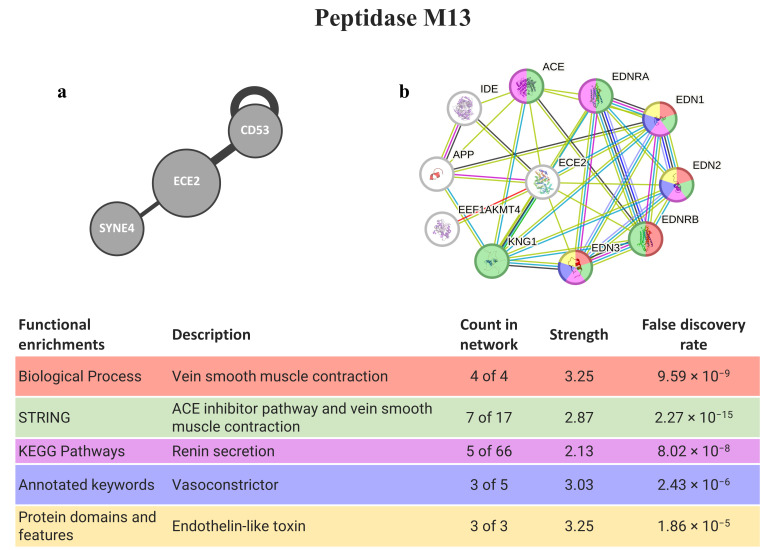
PepM13 interaction pathways for the best homolog matches in humans. (**a**) Human interactome from HuRI for ECE2; (**b**) protein–protein interaction network obtained by STRING software for ECE2. The confidence cut-off for showing interaction links was set to medium (0.400). ACE—angiotensin-converting enzyme, soluble form; APP—gamma-secretase C-terminal fragment 50; CD53—leukocyte surface antigen CD53; ECE2—endothelin-converting enzyme 2; EDN1—endothelin-1; EDN2—endothelin-2; EDN3—endothelin-3; EDNRA—endothelin receptor type a; EDNRB—endothelin receptor type B; EEF1AKMT4—EEF1A lysine methyltransferase 4; IDE—insulin-degrading enzyme; KNG1—kininogen-1; SYNE4—nesprin-4.

Two genes encoding for peptidase M13 were persistently found as the top three hits (ECE2 and MME), but only ECE2 was present in a secretory vesicle membrane (Table 1 and Table 2). In this gene, there are known interactions (from the HuRI) with proteins required for muscle formation/regeneration (CD53) and linkers of the nucleoskeleton and cytoskeleton (SYNE4). Regarding STRING software (Figure 6), several proteins interacting with ECE2 are involved in muscle contraction, and some of them (EDN1, EDN2, and EDN3) contain domains associated with endothelin-like toxin.

**Figure 7:**
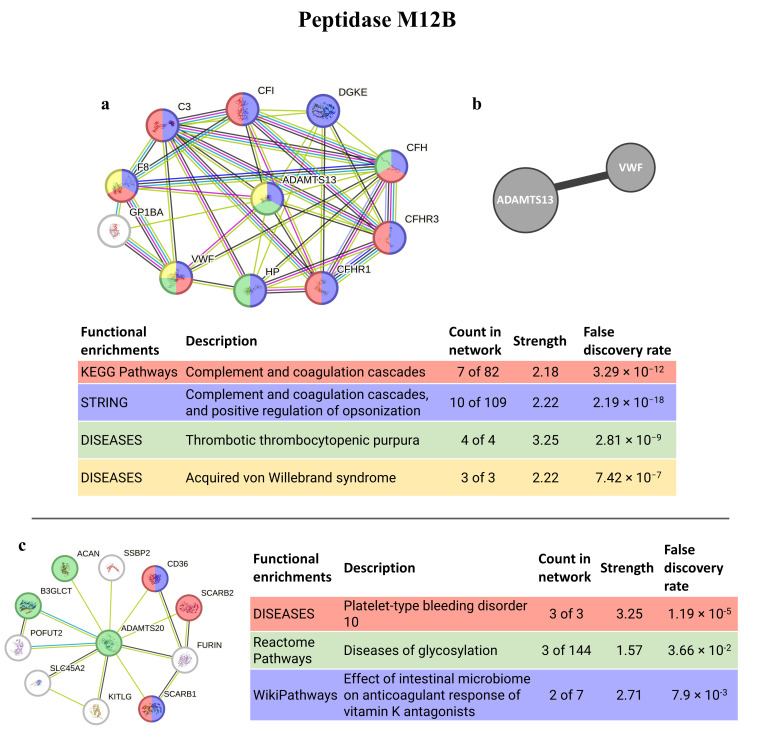
PepM12B interaction pathways for the best homolog matches in humans. (**a**) Protein–protein interaction network obtained by STRING software for ADAMTS13; (**b**) human interactome from the HuRI for ADAMTS13; (**c**) protein–protein interaction network obtained by STRING software for ADAMTS20. The confidence cut-off for showing interaction links was set to medium (0.400). ACAN—aggrecan core protein 2; ADAMTS13—disintegrin and metalloproteinase with thrombospondin motifs 13; ADAMTS20—disintegrin and metalloproteinase with thrombospondin motifs 20; ADAMTSL2—ADAMTS-like protein 2; B3GLCT—beta-1,3-glucosyltransferase; C3—complement C3c alpha’ chain fragment 1; CD36—leukocyte surface antigen; CFI—complement factor I heavy chain; CFH—complement factor H; CFHR1—complement factor H-related protein 1; CFHR3—complement factor H-related protein 3; DGKE—diacylglycerol kinase epsilon; FURIN—Furin; F8—factor VIIIa heavy chain, 200 kDa isoform; GP1BA—platelet glycoprotein Ib alpha chain; HP—haptoglobin alpha chain; KITLG—soluble KIT ligand; POFUT2—GDP-fucose protein O-fructosyltransferases 2; SCARB1—scavenger receptor class B member 1; SCARB2—scavenger receptor class B member 2; SLC45A2—membrane-associated transporter protein; SSBP2—single-stranded DNA binding protein 2; VWF—Von Willebrand factor.

The best homolog match for the peptidase M12B, ADAMTS13, is a protein with stronger connections, interacting mostly with proteins from the extracellular matrix/region (8 out of 10 proteins). It also interacts with the VWF (observed in both software analyses), a protein involved in the maintenance of homeostasis and with blood coagulation abnormalities, such as von Willebrand syndrome and disease, and thrombotic thrombocytopenic purpura. It is also involved in the complement and coagulation cascades and related to hemolytic diseases. The second-best match (ADAMTS20) has weaker interactions, but still has, nevertheless, connections with proteins related to anticoagulative and glycosylation disorders (Figure 7).

## 4. Discussion

The present work was able to successfully isolate and validate the full mRNA coding sequences of six proteinaceous toxins from *Eulalia* sp.: Crisp (phyllotoxin), Hyal, SePr, PepM12A, PepM13, and PepM12B. The ecological relevance of these putative proteins in proboscises, is evidence of its predatorial way of action, which is essential for a marine annelid that feeds on hard-shelled organisms [22]. Plus, the analysis of the related interactions within the human proteome highlighted the relevance of investigating toxins from this species and from other annelids. Moreover, the isolation of the full coding sequences makes them available for future synthesis using recombinant technology.

The analysis of the proteinaceous toxins was compelled by the high overexpression of at least one transcript per toxin from the analysis of the differential gene expression between the proboscis and body wall of *Eulalia* sp., which was previously performed in [21]. The ecological relevance of each putative protein in the cocktail of toxins present in *Eulalia* sp. was seen due to their predatory mode of action and physiological features [34]. This analysis resulted in a selection of different transcripts of interest from the ones selected in the previous work [20], as the intended objective was merely the confirmation of the presence of the toxins mentioned.

These cocktails are the key to effective hunting and predation. The presence of metalloendopeptidases, as well as of serine proteases, is responsible for the degradation of the extracellular matrix [35,36] of *Eulalia*’s preys, while hyaluronidase, with higher homology with hyaluronidases from snakes, acts as a spreading factor in venomous cocktails [37]. Many venomous Crisp-like proteins, on its turn, are known neurotoxins [38], and thus, in this worm, phyllotoxin is thought to act as a muscular blocker capable of paralyzing their prey for a short period of time while the worm feeds [22]. In fact, the homology matching of *Eulalia* Crisp with cysteine-rich venom proteins (CRVPs) from *Conus* [39,40] suggests that phyllotoxin has neurotoxic and paralyzing properties (recall Table 1). These proteins (CRISP and CRVP) are present in a wide range of animals and have been found in the toxins of several invertebrates (see, for example, the cases of snails, marine annelids, and cephalopods [14,21,41,42]). In the case of *Eulalia* sp., the homology matching of proteins from *Homo sapiens* shares similarities with both CRISP2 and CRISP3, which the latter interacts with a receptor with an unknow function (orphan receptor). Even though the interactions within STRING do not highlight any pertinent interaction, the ability to interact with a receptor (HuRI) together with the probable neurotoxic activity of cysteine-rich venom protein (recall Table 1 showing the best hits) highlight its potential in biomedical applications. In humans, both CRISP2 and CRISP3 can be related to male fertility, as it is expressed in the testes and male reproductive tract, respectively (reviewed by Gonzalez et al. [43]), but CRISP3 is mostly associated with the innate host defense, as it can be found as granules in eosinophils, neutrophils, and exocrine secretions [44]. This can be indicative of the potential of this protein as a stimulus for an immune response.

The hyaluronidase from *Eulalia* has higher homology matches with hyaluronidases from highly venomous snakes. In these animals, hyaluronidases have inflammatory actions due to the degradation of products and the generation of hyaluronan fragments, and thus proving the importance of hyaluronidase in envenomation studies [45]. This enzyme shows lower levels of sequence diversity than other toxins across animal taxa [46]. In humans, most hyaluronidases cleave hyaluronan in the extracellular matrix and increases the infusion rates and penetration of molecules, reducing the obstacle that the interstitial matrix presents to fluid and drug transfers [47]. More specifically, HYAL1 and HYAL2 are the major mammalian hyaluronidases in somatic tissues and can act in concert to degrade hyaluronan into a tetrasaccharide [48]. In the case of HYAL4, it has been found to be expressed in placenta, skeletal muscle tissues, and neutrophils. This hyaluronidase does not exhibit hyaluronidase activity (even though is similar in structure with other hyaluronidases), instead exhibiting hydrolytic activity toward chondroitin sulfate chains, degrading them into oligosaccharides [49]. HYAL4 has been noted to be involved in cancer; a defective HYAL4 mechanism may underlie the formation of various cancers, and thus, the swift investigation of HYAL4 in more cancers could provide beneficial insight and a novel, specific treatment target for a variety of cancer patients, including the treatment of acute spinal cord injuries [49,50]. The remaining HYALs, have been used as an adjuvant to increase the absorption and dispersion of injected drugs, to reduce edema in tissues as a healing-promoting agent for skin lesions, and to enhance the local diffusion of anticancer drugs into tissues and tumors (reviewed by Bordon et al. [51]). Furthermore, it may be possible to use a hyaluronidase from bee venom, together with IgG antibodies, to develop novel proteins with reduced immunogenicity to be used as a safer allergen-specific immunotherapy [52]. Hyaluronidases are also directly and indirectly associated with several types of mucopolysaccharidosis, a group of inherited metabolic diseases caused by the absence or malfunctioning of certain enzymes essential for degrading glycosaminoglycans. In the case of mucopolysaccharidosis type IX, it is caused by the deficiency of HYAL1.

Serine protease isolated from *Eulalia* sp. has higher homology than the plasma kallikrein (KLKB1) from mammals, including those from *Homo sapiens* (recall Table 1 and Table 2), involved in the complement and coagulation cascades (recall Figure 4). Plasma kallikrein in humans is the activated form of plasma prekallikrein, a zymogen of trypsin-like serine protease that is predominantly synthesized in the liver. Low levels of this enzyme are found in several extrahepatic tissues. On the other hand, the expression of plasma kallikrein is associated with multiple physiological systems and pathways such as the coagulation pathway, platelet aggregation process, kallikrein–kinin system, renin–angiotensin system, and complement pathway (reviewed by Xie et al. [53]). Moreover, the plasma kallikrein–kinin system (KKS) has been implicated in the pathogenesis of inflammation, hypertension, endotoxemia, and coagulopathy [54]. The balance of KLKB1 is quite tenuous; plasma kallikrein promotes vascular disease and thrombosis in the intravascular compartment, but at low concentrations, it facilitates platelet aggregation induced by adenosine diphosphate, collagen, and adrenaline (reviewed by Xie et al. [53]), but on the contrary, its inhibition may improve cardiovascular disease and thrombosis [53,55]. As such, its inhibition is an effective strategy for the treatment of diseases including hereditary angioedema, microvascular complications of diabetes mellitus, and cardiovascular diseases (see [53,54,56] for further details).

Peptidase M12A (astacins) has different functions, such as promoting the anticoagulation of the blood of prey, venom spreading, and inactivating the vasoactive peptides of prey [57,58]. In *Eulalia*, the best matches are with the zinc metalloproteinases of invertebrates, which, also has homology match with the TLL1/TLL2 of humans, both of which are subfamily members of the metzincin family. TLL1 shares structural similarity with the morphogenetical bone morphogenetic protein-1 (BMP1). TLL1 specifically processes procollagen C-propeptides and cleaves chordin (CHRD gene) at the physiologically relevant site, whereas TLL2 does not exhibit either activity [59]. Both enzymes (TLL1 and TLL2) play a role in tissue remodeling and are upregulated in diverse human diseases, including chronic inflammatory disorders and cancer [60]. These genes, plus CHRD, are amplified in the DNA of specific cancers such as lung squamous cell, and renal and stomach carcinomas, the latter of which are related to poor patient survival with increased CHRD expression [61]. In fact, chordin-like 1 may serve as a potential therapy target via cell cycle regulation and may improve the effectiveness of immunotherapy by regulating immune infiltration [62]. Another CHRD relative, chordin-like 2, has recently been suggested as a clinical biomarker that promotes cell proliferation through the YAP/TAZ pathway in gastric cancer [63].

Homology matching against homologs for peptidase M13 strengthens the homology between invertebrates and humans (recall Table 1), and the certainty of being an endothelin converting enzyme. ECE2 cleaves EDN1 (Endothelin-1) and thus is related to several endothelins and their respective receptors. These endothelins and their receptors are involved in the angiotensin-converting enzyme (ACE) inhibitor pathway and as vasoconstrictors, a potent pro-inflammatory modulator [20] that plays a role in the recruitment of inflammatory cells into tissues by regulating chemokines and adhesion molecules [64,65]. The ACE is involved in the renin–angiotensin–aldosterone system and stimulates the conversion of angiotensin I to angiotensin II [66]; the latter acts as a potent vasoconstrictor that, when inhibited, can reduce blood pressure by dilating vessels and decreasing aldosterone secretion [67]. ECE2 also has known interactions with SYNE4 and CD53 (HuRI software). Nesprin-4, the protein encoded by the gene SYN4, is a kinesin-1-binding protein that displays sun-dependent localization to the outer nuclear membrane. The expression of nesprin-4 is associated with changes in cellular organization, involving the relocation of the centrosome and Golgi apparatus relative to the nucleus and thus may contribute to microtubule-dependent nuclear positioning [68]. CD53 is required for the efficient formation of myofibers in regenerating muscle at the level of cell fusion and may be involved in growth regulation in hematopoietic cells [69]. Apart from the targets of ECE2 retrieved from the interactome-directed analysis related to muscle contraction, Eckman et al. [70] suggested that this peptidase can be involved in the degradation of the peptide β-amyloid, whose accumulation and deposition can lead to the formation of amyloid plaques, a hallmark of Alzheimer disease.

Among all toxins present in *Eulalia* sp., peptidase M12B, also known as reprolysin, has the highest number of transcripts. This toxin has been considered transversal across several clades [71], suggesting the similar permeabilizing capability of this worm, as it occurs in other clades [72]. In fact, the similarities between *Eulalia*’s peptidase M12B and that of marine invertebrates (recall Figure S2) may indicate homology amongst the permeabilizing agents of marine invertebrates. Moreover, previous studies on snake venom metalloproteinases (M12 family), attributes their permeabilizing capability to the degradation of the type IV collagen crosslinked complex network present in the extracellular matrix [73]. Homology matching against human homologs shows similarities with disintegrin and metalloproteinase with thrombospondin motifs (ADAMTS), especially ADAMTS13 and ADAMTS20. The latter has a high conserved physiological role since it was the first with a closely related ortholog in invertebrates; however, it does not have identical zinc-binding active site sequences, and the expression patterns suggests that it may have non-redundant biological roles [74]. ADAMTS13, a gene previously thought to be exclusively from vertebrates [75], cleaves multimers under shear stress conditions and thus controls the size of Von Willebrand factor (vWF) multimers and regulates platelet adhesion, which is mediated by the vWF [76,77]. There are some diseases associated with changes in ADAMTS13. Thrombotic thrombocytopenic purpura (TTP) (recall Figure 7) is a pathological condition that can be characterized by microvascular thrombosis and can occur due to mutations in or the inhibition of ADAMTS13, which might lead to bigger vWF multimers, thus promoting the unregulated formation of platelet thrombi [78,79,80]. Autoantibodies to ADAMTS13 also give rise to acquired TTP [79]. High vWF levels, as seen in stroke and cardiovascular disease, could also be potentially mitigated by recombinant ADAMTS13 more safely than by plasma transfusion [81], thus showing the potential of this protein in biomedical applications.

## 5. Conclusions

The present work successfully integrated ‘omics’ and bioinformatics to isolate the full coding sequence of the most relevant toxins (phyllotoxins) and other bioactive proteins secreted by the species. The evaluation of the properties of these proteins based on protein–protein interactions allowed the identification of potential molecular receptors in the human interactome. We highlight neurotoxins (Crisp), carrying and permeabilizing agents (Hyal), and anticoagulative, coagulative or anti-inflammatory peptides (PepM12A, SePr, PepM12B, and PepM13). Most importantly, the findings also illustrated that combining omics with bioinformatics and legacy molecular methods of sequence isolation and validation can render marine bioprospecting more target-oriented and thus more systematized. The outcomes also enable bioprospecting to be more target-oriented, enabling heterologous expression and more focused bioassays regarding, for example, the choice of in vitro cell lines to test and endpoint.

## Figures and Tables

**Table 1:** Best hits of *Eulalia* sequences against Swiss-Prot database. ADAMTS—a disintegrin and metalloproteinase with thrombospondin motifs; CRVP—cysteine-rich venom protein; mb—membrane; NA—not applicable; ext—external; % ID—the extent to which two (nucleotide or amino acid) sequences have the same residues at the same positions in an alignment, expressed as a percentage.

Toxin	Swiss-Prot Annotated Entries–Best Annotated Matches						
	Protein	Gene	Species	Accession	*e*-Value	% ID	Subcellular Location_Uniprot Annotation
Crisp	Cysteine-rich venom protein TX31	NA	*Conus textile*	Q7YT83	4 × 10^−26^	32.28	Secreted
	Cysteine-rich venom protein Mr30	NA	*Conus marmoreus*	A1BQQ5	9 × 10^−26^	33.06	Secreted
	Cysteine-rich secretory protein 3	*CRISP3*	*Homo sapiens*	P54108	8 × 10^−21^	40.88	Secreted
Hyal	Hyaluronidase	NA	*Crotalus adamanteus*	J3S820	2 × 10^−53^	29.40	Secreted
	Hyaluronidase-2	NA	*Bitis arietans*	A3QVP0	1 × 10^−52^	28.31	Secreted
	Hyaluronidase-1	NA	*Bitis arietans*	A3QVN9	2 × 10^−52^	28.31	Secreted
SePr	Plasma kallikrein	KLKB1	*Bos taurus*	Q2KJ63	7 × 10^−51^	39.93	Secreted
	Plasma kallikrein	*KLKB1*	*Homo sapiens*	P03952	2 × 10^−47^	38.78	Secreted
	Plasma kallikrein	Klkb1	*Mus musculus*	P26262	3 × 10^−46^	36.74	Secreted
Pep M12A	Zinc MP nas-13	*nas-13*	*Caenorhabditis elegans*	Q20191	9 × 10^−42^	27.90	Secreted
	Zinc MP nas-15	*nas-15*	*Caenorhabditis elegans*	P55115	2 × 10^−29^	30.35	Secreted
	Blastula protease 10	BP10	*Paracentrotus lividus*	P42674	5 × 10^−39^	37.66	Cytoplasm; perinuclear region; cell cortex; secreted; ext space
Pep M13	Endothelin-converting enzyme 2	Ece2	*Mus musculus*	B2RQR8	6 × 10^−123^	32.44	Golgi apparatus mb; cytoplasmic vesicle; secretory vesicle mb
	Neprilysin-1	*nep-1*	*Caenorhabditis elegans*	Q18673	1 × 10^−122^	33.48	Mb
	Endothelin-converting enzyme 2	*ECE2*	*Homo sapiens*	P0DPD6	1 × 10^−121^	32.59	Golgi apparatus mb; cytoplasmic vesicle; secretory vesicle mb
Pep M12B	Zinc MP/D	NA	*Bothrops jararaca*	Q98SP2	7 × 10^−12^	23.49	Secreted
	ADAMTS 13	*ADAMTS13*	*Homo sapiens*	Q76LX8	2 × 10^−10^	32.89	Secreted
	ADAMTS 20	*ADAMTS20*	*Homo sapiens*	P59510	2 × 10^−09^	29.05	Secreted; ext space; ext matrix

**Table 2:** Human homologs of the main toxins found in *Eulalia* sp. using only sequences from Swiss-Prot. ADAMTS—a disintegrin and metalloproteinase with thrombospondin motifs; mb—membrane; ext—external; % ID—the extent to which two (nucleotide or amino acid) sequences have the same residues at the same positions in an alignment, expressed as a percentage.

Toxin	Swiss-Prot Annotated Entries-*Homo sapiens*					
	Protein	Gene	Accession	*e*-Value	% ID	Subcellular Location_Uniprot Annotation
Crisp	Cysteine-rich secretory protein 3	*CRISP3* *	P54108	5 × 10^−22^	40.88	Secreted
	Cysteine-rich secretory protein 2	*CRISP2*	P16562	8 × 10^−21^	37.09	Secreted
	GLIPR1-like protein 1	*GLIPR1L1*	Q6UWM5	1 × 10^−15^	32.39	Cytoplasmic vesicle, secretory vesicle, acrosome; cell mb
Hyal	Hyaluronidase-2	*HYAL2* *	Q12891	2 × 10^−47^	27.09	Cell mb: Lipid-anchor, GPI-anchor
	Hyaluronidase-4	*HYAL4*	Q2M3T9	2 × 10^−46^	26.54	Mb: Multi-pass mb protein
	Hyaluronidase-1	*HYAL1*	Q12794	1 × 10^−44^	26.34	Secreted, lysosome
SePr	Plasma kallikrein	*KLKB1*	P03952	1 × 10^−48^	38.78	Secreted
	Transmembrane protease serine 3	*TMPRSS3*	P57727	9 × 10^−46^	37.59	Endoplasmic reticulum mb
	Prostasin	*PRSS8*	Q16651	8 × 10^−44^	34.69	Cell membrane
Pep M12A	Tolloid-like protein 1	*TLL1*	O43897	3 × 10^−33^	28.84	Secreted
	Tolloid-like protein 2	*TLL2*	Q9Y6L7	8 × 10^−31^	29.67	Secreted
	Bone morphogenetic protein 1	*BMP1*	P13497	1 × 10^−30^	33.00	Golgi apparatus; secreted, ext space, ext matrix; secreted
Pep M13	Endothelin-converting enzyme 2	*ECE2* *	P0DPD6	7 × 10^−123^	32.59	Golgi apparatus mb, cytoplasmic vesicle, secretory vesicle mb
	EEF1AKMT4-ECE2 transcript protein	*EEF1AKMT4-ECE2*	P0DPD8	4 × 10^−122^	32.59	Golgi apparatus mb, cytoplasmic vesicle, secretory vesicle mb
	Neprilysin	*MME*	P08473	2 × 10^−115^	30.76	Cell mb
Pep M12B	ADAMTS 13	*ADAMTS13* *	Q76LX8	1 × 10^−11^	32.89	Secreted
	ADAMTS 20	*ADAMTS20*	P59510	1 × 10^−10^	29.05	Secreted, ext space, extmatrix
	ADAMTS 1	*ADAMTS1*	Q9UHI8	2 × 10^−09^	27.01	Secreted, ext space, ext matrix

* Proteins found in the HuRI (the Human Reference Interactome).

## Data Availability

Isolated sequences are also publicly available at GenBank under the accession numbers OP254189–OP254194.

## Supplementary Materials

The following supporting information can be downloaded at: https://www.mdpi.com/article/10.3390/ani14040635/s1.
