# Supplementary material for: Exploration of Toxins from a Marine Annelid: An Analysis of Phyllotoxins and Accompanying Bioactives
Source: Animals (Basel). 2024 Feb 16;14(4):635. doi: 10.3390/ani14040635 (PMC10885894; doi:10.3390/ani14040635)
Supplement: Supplementary file 1 [file animals-14-00635-s001.zip › animals-2822341-supplementary.pdf]

Table S1. Primer sequences used for validation of sequences obtained by RNA-seq.

Target	Forward (5’-3’)	Reverse (5’-3’)	Amplicon Size (bp)
Pep M12A (AP1)	TATGTTGTATGTCCATCCGA	GGTGTCTTGTCACATTCTC	1045
Pep M12A (AP2)	AGAAGGTTACTTGGACTCG	CGGACAACTGTTCGTTTTAT	511
SePr (SP1)	TTCTTGTCTGTTCCAGTGAA	GCCCGATATGTGCTGTATAA	819
SePr (SP2)	GGAGCATCTCAGGAAGAATC	GTGTAGTTCAGGGTGTGATT	934
SePr (SP3)	TTCATCCGCAGTATGTTCTC	TCCGACCGTCTTCTCTATAC	867
Pep M13 (MP1)	TTAGTTCCTGCGTGCATC	AGCCAACTTGACTTTCTTCA	773
Pep M13 (MP2)	CCAGAGGTTGAAAGATGTAGTT	CGCTGTATCGGAGAAATACT	912
Pep M13 (MP3)	CGTCGATAGTTGGTATCCTC	CCTTCTTTCAACCCTCCATT	842
Pep M13 (MP4)	TGACACTTGCGGAAAACAT	GGAGAATCCCTGGTTAAGG	398
Pep M12B (RP1)	TAAGGATATGTTGCTTGCCTC	TCTGTTTGGTCGTCTTAGTG	700
Pep M12B (RP2)	GAACATCACCCTCACATCAT	TCTCGGCATAAACTGACAAA	1129
Hyal (HP1)	GCACAAAGGTCTCTTCCA	CGAGATGTCTTTACAGGTTCT	721
Hyal (HP2)	ACTCTGCAACAGCCCTAA	ACAGCGGCACTTGTATTC	594
Crisp	CGGAATCTCCTGTTAGAATGAT	ATTCAAGTAACATAATTTCCTCTGAC	727

Table S2. Primer sequences used for expression validation by RT-qPCR.

Target	Forward (5’-3’)	Reverse (5’-3’)	Amplicon Size (bp)
Pep M12A	AGAAGGTTACTTGGACTCG	CAGCAGGCATATCTCATGTA	148
Pep M12B	AAGGATATGTTGCTTGCCT	AGATGACGAAGGTACACG	168
Pep M13	TGACACTTGCGGAAAACAT	GCCAGATACGAGTCCAAA	191
SePr	GACCACAGAATATGCCAAGA	CTCAGGTCACCTTCATTCAC	200
Hyal	GCACAAAGGTCTCTTCCAG	CAGGGGCTGTGTTTAGTG	217
Crisp	GAATGATGTTCTCCGTGGC	ATCTTTAGCCTTGGGCGT	179
18S	CGATGGTACGTGATATGCC	CGAATGAGTCCCGTATTGT	176

The data obtained from transcriptomic was confirmed by Sanger sequencing. After sequence confirmation, expression was also accessed.

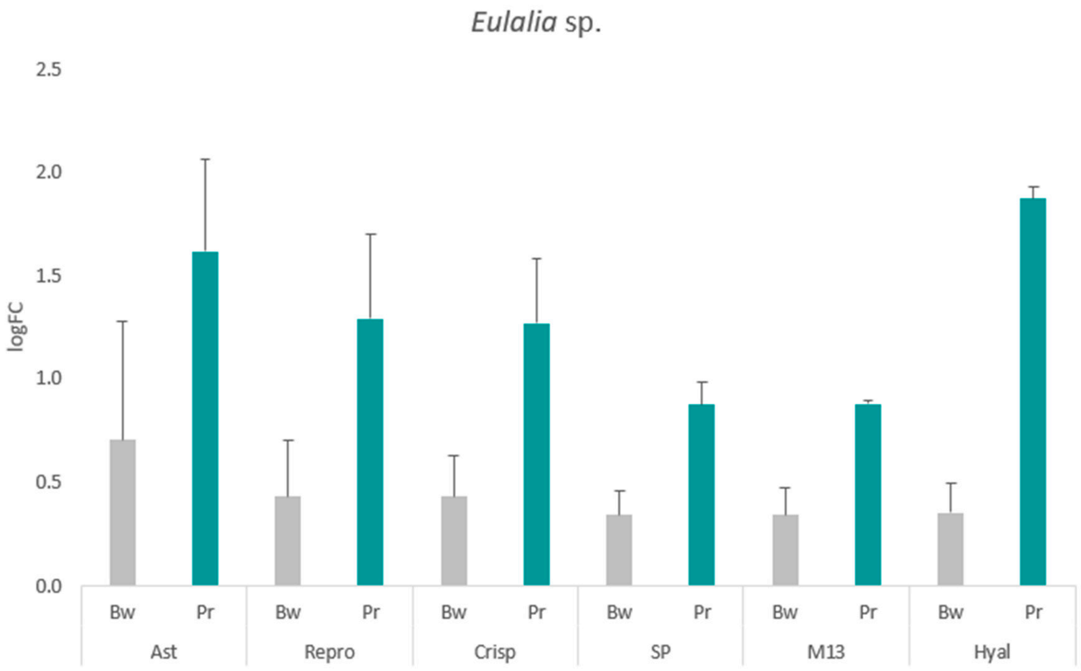

Figure S1. Expression analysis of key toxins by RT-qPCR, comparing the proboscis and body wall. Data are expressed as mean relative expression of Crisp (cysteine-rich venom protein), Pep M13 (Peptidase M13), Hyal (hyaluronidase), SP (Serine protease), Repro (peptidase M12B) and Ast (astacin, peptidase M12A). The housekeeping gene 18S was used for normalization. * Indicates significant differences to body wall for each respective target (Kruskal-Wallis H, $p < 0.05$).

Table S3. Protein predicted Open Reading Frames for the putative protein of interest identified by RNA-seq and validated by Sanger sequencing.

Protein	Accession	Size	Open Reading frame
Crisp	OP254189	997	MMFSVAAVLMPCLLLAAGAEIGELQTLLRGARGVSDADKKTIVDRHNELRAAVSPKAKDMTKMYWNDAIAAGAQQWVDTCPNGHDTNWDRMKNAGLW<br>ISIGQNWAGGYSDWTAAINGWHSEVSNFELGQGSTNGQAVGHYTQVVHNDAIAVGCAFGDCSGSKIYVCNYAYGQMDYKTPYGDGGSSSCGDCSGKC<br>SNNLCDCGAKVCQNGGTMKLSDCTCSCKSFFSGDQCETKDCSQEDPGYCTSSYGESACQTYGNVPTDCPHMCGVC-
Hyal	OP254194	1254	MAPPALHVAFALAINIVTIVAEEPFQVIWHAPTEDKWGCGQKYHIDIDYASYDLTLNTAPGSDGWYKGDVITIMYVDELWPHLKGSDNFADDYERVY<br>GGLPQSGDIDADFDRLAKLIEEKIPNKEFAGLAVLDFEKWRPLYDWNFKNGILGRYQRISRKKVEEDRKGENLSAEEIEDIARQEFDDAARSYFEGA<br>LIMAKGMRPNAKWGYYIYPLCNSPNLERTCKDISYDAIKKLKWMVDASGALFPSAYFEEHMSVREQRPYIYGANSISLDWVKMSNDTSKPIYTYNAI<br>VKPMSQYGPKFIFYDREALVNGIGICADMGMNGVPMWGNGNLRNRTAACEEVKEWVEGTFGPYVKRVVEFTKKCSEDLCSSQGRCYKENIEKDSDKV<br>DYSAESSHVYDADKVKEEYKCRCYEGWKGDSCDQQV-
SePr	OP254193	599	MGTYTISLLGIFAVLPCIASLPNNCHDQLGVANTGLFPKYWLLSSDGDAYRGRLQQDPAWLSELPTYDDQNRTQPFAWIQTDLHDLTVVSGVLTQGA<br>SQEESWVSHFSVWSSRDCENFSPMLDSDGDMAVFHGNYDRSTIVTTIFHDILVTRCVRIVPIETVGNHTALRLELLGCDHRICQEEVIQHISGDGMR<br>QNGSASTSLPVEISHQATLVRSGFAEFQLPSEHVVFGIVFYVNEGDLSRNSRAIITYSRNCHTWEPIREKKGYGDPKVFNLGAGKFRSKQWSFPYPV<br>RAHCLRLIPSTSRFTDENSAQANFIGCGTGEGVIWQCGQQRPVTSLRRRKRIVGGTPSGLGWWPWLASLRIRYPDNPDFEHVCGGTLVHPQYVLTAA<br>HCMNVIYSNLLQNHTLNYTLWNDPEMLENMVVRLGEYRLDDTEGTQVDLHVLEIVAHPGYNISYYIKDVALLRLERPLRLTDHINTACLPQSNHAFS<br>DHQVCQVIGWGTTGIAMPARPYHVAIPLVPQDECQKLYAHLLITPDMLCAAAPEGGKDACMGDSGGPLVCERDSQWYLVGVVSWNIGCGRRGYPGVY<br>ARVPFFLDWLEEVMGTL-
Pep M12A	OP254190	424	MDFHVFLFSVFVVWQTSHGKTIDQTILSSLPRGQDGNITTSLKDAALLELDIRVSHEEYETLKSQPFSKRKATPHENRRWTDNTIPYTIGTGFNDQE<br>KKHVRTAMDEWERYTCLKFRDATPADTNMVKIESGEGCNAGIGMMGGAQILNLGVNCKMVSVALHELGHIIGLYHEQNRRDRDDYIWVRLEHMNPTY<br>RFAAEKTKEGDIDNYGVPYDYGSIMHYGLDAFASGKEPTIITNDPTWQFRIGNARHLSFNDIKIVNIIYKCGSRCGGKTCPGEGYLDSNCQCMCPGN<br>PTRPCSSPAPEVDPCVDFDTSCPGHAAVGECDKTPGYMRYACWSSCQMCGEGCKDKQADCLTRARKGDCKGNPIYMLDNCWKSCGFCFGKGNEPSAS<br>CQDTNPKCGLWAAQGQCVTHTDYMQMGCPRACAFCN-
Pep M12B	OP254191	555	MAFPWLCALLALVGIVCGTPVERKRDLHGKRVYLRHLTVAQAKSLMQGNGTMMPDGIDPMADGIDPMADGIDPMADGIDPEHLDDTPEIMPVTMHTE<br>EGGHIHGLLLQRNFRVEHPVTVSFNDGVTETENVPLNAEGHAQYQDSMSSGVFGVRHVDGRKHFDGHFYLDGENYEVHPLAEDDGVRARGFEEHHPH<br>IITRTEPLNYSDPIDLKDEHVRVTKTTKQKRGGHLDVEMAIVIDYGYYLRWLEKMGSQGAAVSEIKYMFSFNYNGIDSIYKTVNYQGMTITPWLKAL<br>HIQTSSNGNLALQLNPGINPSGGRSNVVDGEQILDKMAESWGPTNGFGVPSNYDTVMTYVGYKISRNGDPKVIGIAFTGHVCSPYGYSIVEDQGFNT<br>FKVAAHELGHTLGAKHDVEPSGDCSPGKHHIMESQDAMPNEANNMDGYTFSTCSLARMLEYTGRATCLGNRGSNSFGPVPVSPLPGEKFSASEQCLW<br>YHSWQNPSSYHCRGSKATVSAEECWRGLWCSNNPTNCQRWENLRAAEGTPCAGGKKCIHAQMSIEQCLRI-
Pep M13	OP254192	665	MLQWMNVSQDPCEDFYLYSCGNYIANQKIPEGQLYWNFISMFTEVLNQRLKDVVDVEIEPSDMDSVKKVKLAYQACMDQEAQNALGGQPMLDVLDTL<br>GTWPPLNPSWDESSVDIATLMAQVMLLGRQTYATLTVKANSSDNTHNAIYMDSPGIAMAAYLDSDAAEAQPVVASYRQIVLEMALLLVNNNPADRNF<br>LETEINTMIDFEAKLANITLQNTGVSYILSLAELKVEYPQIWDWDDYLNAYLAPSGRSLSDSEVMTMYNMEYYGLLKNLLDNTSKRTIVTYLLWNVV<br>DSWYPLLAKEFRDLSDRKLALLYPIASAPPQWQYCVSVANGYLPAVSGRLYVDQYFSNTAKVDVEMIGEDLKTTLFSVLEEASWMDATTMALAIEKL<br>ENMPIKVGYPPFIMDDEALNQEYEGVDITNATQFMNLVHISQHQVNSLVKNYYVPIDLDAWSMTATTMNAAYIYNRNAVEICAAILQQYAFYQDYPR<br>FMNYGGIGVFVGHELTHGFDSIGRNFDLHGNIQSWWTAETEQVFYERAGCIVNQYSGFTFPGTDITVNGMLTLAENIADNGGLKEGYIAYQSWLERN<br>GGEEPSLPGFDHMSNEQMFFLSWSQLWCEVSLDSYLANQVVTNNHAPNRYRVIGTLQNSPDFARVYNCPAGSYMNPETKCEVW-

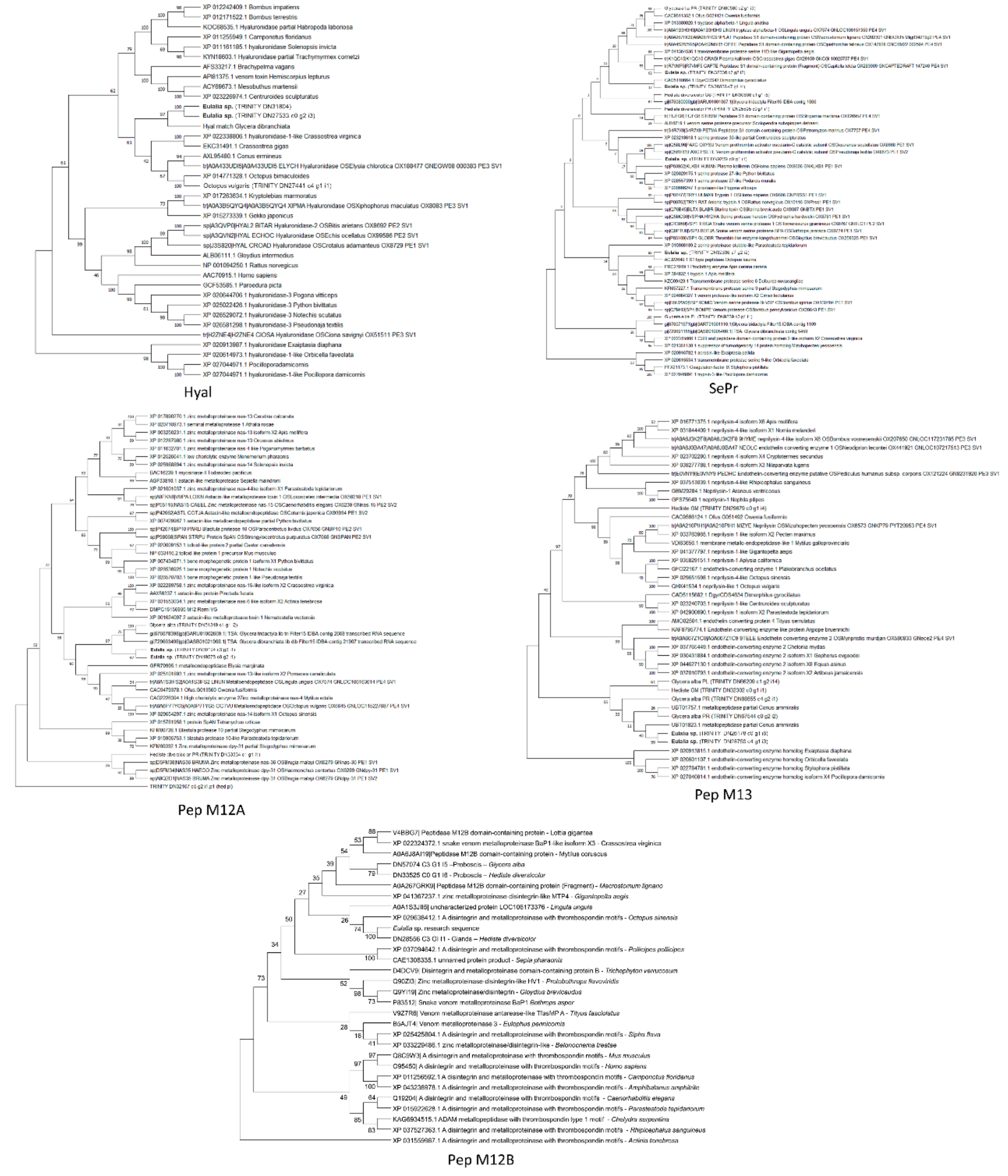

Figure S2. Phylogenetic trees of the short-listed toxins secreted by *Eulalia* sp. The models include the validated sequence in the worm and the best matches from all animals for comparison. The phylogenetic reconstruction was made with MEGA X, with 1000 bootstrap pseudoreplicates. Bootstrap support values are given for all nodes and clade names are indicated by colored branches.
